# Cell subtypes and immune dysfunction in peritoneal fluid of endometriosis revealed by single-cell RNA-sequencing

**DOI:** 10.1186/s13578-021-00613-5

**Published:** 2021-05-26

**Authors:** Gen Zou, Jianzhang Wang, Xinxin Xu, Ping Xu, Libo Zhu, Qin Yu, Yangying Peng, Xinyue Guo, Tiantian Li, Xinmei Zhang

**Affiliations:** Department of Obstetrics and Gynecology, Women’s Hospital, School of Medicine, Zhejiang University, 1 Xue Shi Road, Hangzhou, 310006 Zhejiang People’s Republic of China

**Keywords:** Endometriosis, Single-cell RNA-sequencing, Peritoneal fluid, Cell profiling, Immune dysfunction

## Abstract

**Background:**

Endometriosis is a refractory and recurrent disease and it affects nearly 10% of reproductive-aged women and 40% of infertile patients. The commonly accepted theory for endometriosis is retrograde menstruation where endometrial tissues invade into peritoneal cavity and fail to be cleared due to immune dysfunction. Therefore, the comprehensive understanding of immunologic microenvironment of peritoneal cavity deserves further investigation for the previous studies mainly focus on one or several immune cells.

**Results:**

High-quality transcriptomes were from peritoneal fluid samples of patients with endometriosis and control, and firstly subjected to 10 × genomics single-cell RNA-sequencing. We acquired the single-cell transcriptomes of 10,280 cells from endometriosis sample and 7250 cells from control sample with an average of approximately 63,000 reads per cell. A comprehensive map of overall cells in peritoneal fluid was first exhibited. We unveiled the heterogeneity of immune cells and discovered new cell subtypes including T cell receptor positive (TCR+) macrophages, proliferating macrophages and natural killer dendritic cells in peritoneal fluid, which was further verified by double immunofluorescence staining and flow cytometry. Pseudo-time analysis showed that the response of macrophages to the menstrual debris might follow the certain differentiation trajectory after endometrial tissues invaded into the peritoneal cavity, that is, from antigen presentation to pro-inflammation, then to chemotaxis and phagocytosis. Our analyses also mirrored the dysfunctions of immune cells including decreased phagocytosis and cytotoxic activity and elevated pro-inflammatory and chemotactic effects in endometriosis.

**Conclusion:**

TCR+ macrophages, proliferating macrophages and natural killer dendritic cells are firstly reported in human peritoneal fluid. Our results also revealed that immune dysfunction happens in peritoneal fluid of endometriosis, which may be responsible for the residues of invaded menstrual debris. It provided a large-scale and high-dimensional characterization of peritoneal microenvironment and offered a useful resource for future development of immunotherapy.

**Supplementary Information:**

The online version contains supplementary material available at 10.1186/s13578-021-00613-5.

## Background

Endometriosis is a chronic inflammatory disease and affects nearly 10% of reproductive-aged women and as high as 40% of infertile women [1]. It is clinically manifested with severe pelvic pain and reduced fertility and is characterized by the presence and growth of endometrial tissue outside the uterus, which seriously reduces the life quality of patients and causes a heavy burden on the healthcare [2, 3]. The etiology is still incompletely understood and “retrograde menstruation” is one widely accepted theory that the reflux accounts for the accumulation of menstrual debris in peritoneal cavity [4, 5]. However, retrograde menstruation occurs in almost all cycling women, while only a minority of them develops endometriosis, implying that additional factors contribute to the development of endometriosis [6].

Immune cells contribute to scavenging menstrual debris in cycling women [7]. One of the possible causes of endometriosis is the defective immune response to the refluxed menstrual debris in peritoneal cavity, which determines the survival and implantation of ectopic endometrial cells and lesion formation [8]. Accumulated evidence over the past decade has suggested that development of endometriosis is accompanied with sustained peritoneal inflammation, including altered immune cell contents in peritoneal fluid and ectopic lesions, as well as changed immune cells cytotoxicity and activation [9]. Alterations in both innate and adaptive immunity contribute to the pathogenesis of endometriosis [9–11]. As the front line of innate immunity, macrophages comprise the largest immune cell population in peritoneal fluid of both healthy women and endometriosis patients [12]. They are complex cells at the center of this elusive condition, which are critical for the growth, vascularization and innervation of endometriosis lesions. Previous studies have demonstrated functional disorders of macrophage in endometriosis. However, it is still controversial whether macrophages in peritoneal fluid exhibit a pro-inflammatory or pro-repair phenotype [13]. Recent studies have revealed that they are complex and heterogeneous in the pathology of endometriosis [14]. In addition to macrophages, other immune cells also have been proposed to play important roles in the pathogenesis of endometriosis. Decreased cytotoxicity of natural killer (NK) cells had been reported in peritoneal fluid of endometriosis [15]. An increased proportion of regulatory T (Treg) cells in peritoneal fluid of women with endometriosis has been reported [16]. Dendritic cells (DCs), mast cells and B cells have been observed to be changed as well [17–19].

Previous studies mainly focus on one or several immune cells and are lack of comprehensive investigation, which leads to a failure to discover heterogeneous cell contents at an unbiased scale. Recent advance in single-cell RNA-sequencing (scRNA-seq) has the potential to resolve heterogeneous cell populations at an unprecedented scale [20]. It has been used to discriminate cell types in healthy tissues or tumors, to explore immune cell heterogeneity and to reveal new types of immune cells [21, 22]. Since peritoneal fluid plays an important role in the pathogenesis of endometriosis, it warrants an unbiased characterization of cell contents and scRNA-seq of all the cells in peritoneal fluid. Here, we found that the peritoneal microenvironment is mainly composed of different immune cells. We further found that the immune cells in endometriosis were dysfunctional with decreased phagocytosis and cytotoxic activity and elevated pro-inflammatory and chemotactic effects. Importantly, our findings offer a useful resource for understanding pathology of endometriosis and potential immunotherapy of endometriosis.

## Methods

### Ethics and sample collection

This project was approved by the Ethics Committee of Women’s Hospital, School of Medicine, Zhejiang University (IRB-20200003-R). Included patients supplied written informed consent for collection of specimens and analyses of the derived genetic materials prior to their participation.

Peritoneal fluid samples were collected from 39 endometriosis patients and 27 non-endometriosis controls undergoing surgery (details in Additional file 2: Table S1). All included patients did not receive hormone treatment in the 6 months and all samples were collected in proliferative phase. Among them, cell suspensions from one endometriosis patient and one control patient with septate uterus were subjected to scRNA-seq and the other 64 samples were applied for the validation using double immunofluorescence or flow cytometry. Samples were collected during laparoscopic surgery before any surgical procedure to avoid contamination from blood. Samples were transported to the laboratory in a cold chain within 30 min and used for subsequent experiments.

### Cell preparation

Cells were pelleted from peritoneal fluid and washed three times. Red blood cells were lysed using Ammonium-Chloride-Potassium Lysing Buffer (Gibco, USA) according to the manufacturer’s instructions. Samples were next diluted with PBS containing 0.04% Bovine Serum Albumin (Sigma, USA) to the density of about 1 × 10^6^ cells/mL. 10 µL of this cell suspension was mixed with 10 μL 0.4% trypan blue solution (Sigma, USA) and counted using an automated cell counter (Bio-rad, USA) to determine the density of live cells. Cell viability of samples used for single cell sequencing was 94% for endometriosis sample and 86% for control sample. Cells were maintained on ice whenever possible throughout the dissociation procedure, and the entire procedure was completed less than one hour.

### scRNA-seq using 10 × genomics

The density of single cell suspension was counted and adjusted to 1000 cells/μL. The cell suspension was loaded into Chromium microfluidic chips with 3ʹ (v3) chemistry and barcoded with a 10 × Chromium Controller (10 × Genomics) in order to catch approximately 10,000 cells/chip position. The remaining procedures including reverse transcription and the library construction were performed according to the standard manufacturer’s instructions. Single cell libraries were sequenced on NovaSeq with approximately 50,000 to 100,000 reads per cell. Single-cell analyses were performed using Cell Ranger 3.0 and Seurat unless mentioned specifically. For the quality control, low quality cells (< 3 cells/gene, < 200 genes/cell, > 6 500 genes/cell, > 5% hemoglobin genes and > 30% mitochondrial genes) were removed. The average gene detection, number of UMIs and the level of mitochondrial reads were similar between the two samples (Additional file 3: Table S2).

### Identification of cell clusters by UMAP analysis of scRNA-seq datasets

To identify major cell types, we performed uniform manifold approximation and projection (UMAP) clustering using the Seurat package of R software. To determine the cell types, we used a combination of marker genes identified from the literature and the web-based CellMarker databases (http://biocc.hrbmu.edu.cn/CellMarker/). The detailed marker genes were listed below: protein tyrosine phosphatase receptor type C (PTPRC/CD45) for leukocytes, CD68 molecule (CD68), CD14 molecule (CD14) and Fc fragment of IgG receptor IIIa (FCGR3A/CD16) for macrophages, integrin subunit alpha X (ITGAX/CD11C), major histocompatibility complex, class II, DR alpha (HLA-DRA) and CD1C molecule (CD1C) for DCs, CD3D molecule (CD3D), CD3G molecule (CD3G) and CD3E molecule (CD3E) for T cells, killer cell lectin like receptor D1 (KLRD1), natural killer cell granule protein 7 (NKG7) and killer cell lectin like receptor B1 (KLRB1) for NK cells, immunoglobulin heavy constant mu (IGHM) for plasma cells, carboxypeptidase A3 (CPA3) and GATA binding protein 2 (GATA2) for mast cells, ITGAX, CD1C and KLRB1 for NKDCs, keratin 8 (KRT8) for epithelial cells, solute carrier organic anion transporter family member 5A1 (SLCO5A1) and NCCRP1, F-box associated domain containing (NCCRP1) for multilymphoid progenitor cells. Heatmaps and violin plots were generated from R package using the default complete-linkage clustering algorithm.

### Biological process enrichment analysis, pathways analysis and single cell trajectories

We used the web-based DAVID and KOBAS to perform biological process enrichment analysis with the differentially expressed genes in each cluster. Gene Set Enrichment Analysis (GSEA) was applied to identify a priori defined set of genes that show differences in each cell types between endometriosis and control samples. We used the mean expression of genes in endometriosis and control samples as the input (Additional file 4: Table S3), and implied gene sets of KEGG pathways (http://software.broadinstitute.org/gsea/downloads.jsp#msigdb), which were corrected in Molecular Signatures Database (MSigDB). The Monocle package of R software was used to analyze the single cell trajectory in macrophage subtypes in order to discover the developmental transition of macrophages.

### Antibodies

Pre-conjugated antibodies were purchased from eBioscience, Abcam, Santa Cruz and BD company. The detailed information including working volume was listed in Additional file 11: Table S10. Cell surface and intracellular staining were performed according to the manufacturer’s recommendations.

### Double immunofluorescence staining

For the staining of membrane proteins, including CD14 (eBioscience, USA), TCR Cβ1 (Santa, USA), CD1C (eBioscience, USA) and KLRB1 (eBioscience, USA), ascites cells were incubated with the indicated fluorochrome- or biotin-conjugated antibodies. For the staining of KI67 (BD Biosciences, USA), 1 × 10^6^ cells were firstly resuspended in 250 µL BD Cytofix/Cytoperm solution and then incubated with 20 µL antibody. The nuclear was stained by DAPI and the samples were analyzed with FV1000 confocal microscope.

### Flow cytometry

Flow cytometry was conducted to measure the percentages of CD14^+^ TCR Cβ1^+^ and CD14^+^ KI67^+^ cells and to sort CD14^+^ and KLRB1^+^ KLRD1^+^ cells in peritoneal fluid. For cell surface staining, the cell suspension was incubated with CD14 (eBioscience, USA) and TCR Cβ1 antibodies. For CD14 and KI67 staining, the cell suspension was incubated with CD14 antibody at 4 °C for 30 min, and then incubated with the BD Cytofix/Cytoperm solution. After wash, the cells were incubated with 20 uL KI67 antibody for 30 min at 4 °C. The suspension was centrifuged, washed and re-suspended with 500 μL PBS to detect the positive cells with Cytoflex S Flow Cytometer. The results were analyzed with CytExpert in percentage. For cells sorting, the cells were incubated with CD14 (Abcam, USA), KLRB1 (eBioscience, USA) and KLRD1 (eBioscience, USA), and the cells were sorted by Beckman moflo Astrios EQ.

### Phagocytosis tests

pHrodo™ Red E. coli BioParticles™ Conjugate (Invitrogen, USA) was used to perform phagocytosis tests for macrophages. After initial cell preparation, each sample was mixed sufficiently and divided into two tubes with approximately 1 × 10^6^ cells per tube. One sample was incubated with 20 µL CD14 (Abcam, USA) for 45 min at 4 °C for background. The other sample was first incubated with 20 µL CD14 for 45 min at 4 °C, and then 100 µL pHrodo™ Red E. coli BioParticles™ (1 mg/mL) was added. The suspension was resuspended and incubated for 1.5 h at 37 °C. Cytoflex S Flow Cytometer was used to detect the mean fluorescence intensity of CD14 + macrophages with ingested bioparticles.

### Quantitative real-time PCR

Total RNA was extracted from cells with TRIzol reagent (Invitrogen, USA) and reversed using a PrimeScript Reverse Transcription (RT) reagent kit (Takara, Japan) according to the manufacturer's recommendations. Specific primers used for amplification were listed in Additional File 12: Table S11 (Sangon Biotech, China). Real-time PCR was performed with an Applied Biosystems 7900HT system (ABI, USA) using a SYBR Premix Ex TaqTM kit (Takara. Japan). An average cycle threshold (Ct) value was calculated from triplicate wells for each sample, and the fold change was determined by the 2^−△△Ct^ method.

### Statistical analysis

Data were presented as mean ± standard error of the mean (SEM). Independent-sample t-test or Mann–Whitney U-test was applied when comparing two samples, and One-way ANOVA or Kruskal–Wallis was employed when comparing 3 or more samples. Statistical difference was considered to be significant at a value of *P* < 0.05 (*), highly significant at a value of *P* < 0.01 (**) and extremely significant when *P* < 0.001 (***) or *P* < 0.0001 (****). Differential gene expression testing was performed in Seurat as described in the scRNA-seq section.

## Results

### Single-cell expression atlas and cell types in peritoneal fluid

**Fig. 1:**
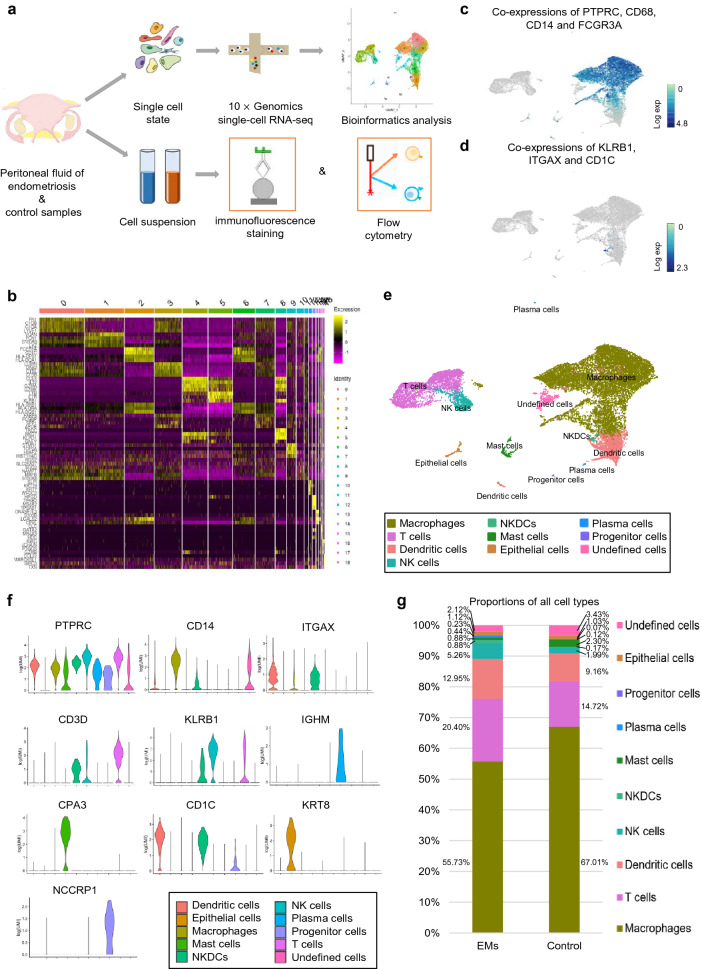
Cell profiling in peritoneal fluid delineated by single cell transcriptomic analysis. **a** The workflow that depicts the collection and processing of peritoneal fluid from endometriosis and control patients for single-cell RNA-sequencing and further study. **b** The heatmap exhibited that the differentially expressed genes of 17,530 isolated cells gathered in 19 clusters. **c** Co-expressions of PTPRC/CD45, CD68, CD14 and FCGR3A/CD16 (macrophage makers) on the UMAP plot. **d** Co-expressions of KLRB1, ITGAX and CD1C on the UMAP plot. **e** 2D visualization of ten cell types on the UMAP plot. Each dot corresponds to one single cell. **f** Violin plots of specific marker genes in all cell types. **g** Bar plot of proportions of all cell types in the endometriosis and control samples. *EMs* endometriosis, *NK cells* natural killer cells, *NKDCs* natural killer dendritic cells

To explore the cell profiling in peritoneal fluid, scRNA-seq was performed (Fig. 1a). After initial quality control (Additional file 1: Figure S1a), we acquired single-cell transcriptomes in a total of 10,280 cells from endometriosis sample and 7250 cells from control sample with an average of approximately 63,000 reads per cell (Additional file 3: Table S2). Cell transcriptomes from the two samples were merged and analyzed together to gain power to detect rare cell types. To explore the intrinsic structure and potential functional subtypes of overall cells in peritoneal fluid, we applied principal component analysis (PCA) with variable genes across all cells and identified 19 clusters (Fig. 1b, Additional file 1: Figure S1b and Additional file 5: Table S4). There were no unique populations identified in either dataset including endometriosis sample and control sample. We then used well-known marker genes to define the identity of each cell cluster (see “Methods”), such as co-expression of PTPRC/CD45, CD68, CD14 and FCGR3A/CD16 for macrophages (Fig. 1c) [23, 24]. Cluster 15 expressed marker genes for DCs (CD1C, ITGAX/CD11C) and NK cells (KLRB1) (Fig. 1d), and it mostly matched with natural killer dendritic cells (NKDCs), a rare intermediate cell type reported by Pillarisetty et al. [25]. Eventually, we identified 9 main cell types (Fig. 1e), including macrophages (clusters 0, 1, 3, 6, 7, 9 and 11), T cells (clusters 4 and 5), DCs (clusters 2 and 14), NK cells (cluster 8), epithelial cells (cluster 12), mast cells (cluster 13 and 16), NKDCs (cluster 15), plasma cells (cluster 17) and multilymphoid progenitor cells (cluster 18). Otherwise, there was one cluster (cluster10) that we failed to match with any cell type because it lacked recognizable maker genes. Violin pictures and UMAP plots for each cell type further supported these cell types (Fig. 1f and Additional file 1: Figure S1c). Eventually, we captured a comprehensive map of overall cells in peritoneal fluid (Fig. 1e, g and Additional file 6: Table S5). Immune cells predominated in peritoneal fluid of both endometriosis (96.5%) and control (95.5%) groups, and macrophages were the main immune cells, followed by T cells, DCs, NK cells, mast cells, epithelial cells, NKDCs, plasma cells and multilymphoid progenitor cells, which indicated that peritoneal cavity was an immune microenvironment.

### Distinct subtypes of macrophages revealed the heterogeneity of macrophages in peritoneal fluid

**Fig. 2:**
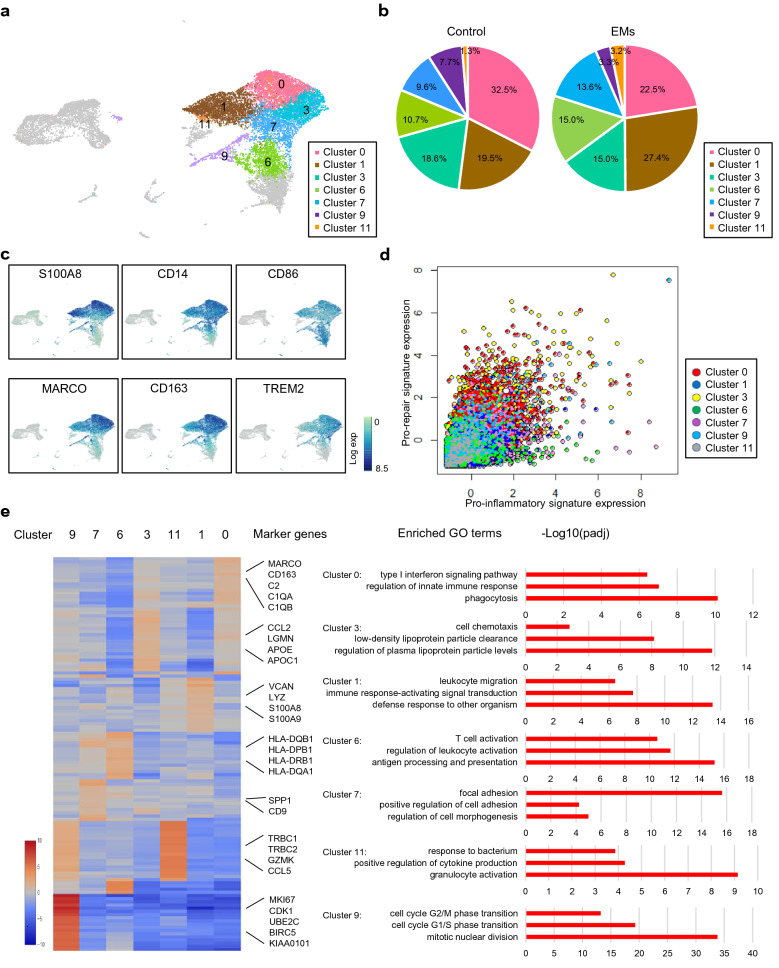
The heterogeneity of macrophages in peritoneal fluid. **a** 2D visualization of seven clusters of macrophages on the UMAP plot. **b** Pie charts of proportions of seven macrophage subtypes in endometriosis and control samples. **c** Distributions of pro-inflammatory (S100A8, CD14 and CD86) and pro-repair (MARCO, CD163 and TREM2) marker genes on the UMAP plots. **d** Scatter plot of normalized gene expression of pro-inflammatory and pro-repair signatures per cell. Each dot corresponds to one single cell. **e** Heatmap, marker genes and enriched GO terms of seven subtypes of macrophages. *EMs* endometriosis

We identified 7 clusters representing different subtypes of macrophages (Fig. 2a). The proportions of each subtype of endometriosis and control groups were exhibited in Fig. 2b. Firstly, we investigated the pro-inflammatory/pro-repair polarization paradigm and found that one cell could express both pro-inflammatory and pro-repair marker genes, such as the high expression of macrophage receptor with collagenous structure (MARCO) and S100 calcium binding protein A8 (S100A8) in cells of cluster 0 (Fig. 2c). Scatter plot further revealed that pro-inflammatory gene signatures were correlated with pro-repair gene signatures and there was no significant shifting from pro-repair to pro-inflammatory or from pro-inflammatory to pro-repair (Fig. 2d). These findings supported the idea that macrophages in peritoneal fluid did not comport with the pro-inflammatory/pro-repair polarization model and the simplified view of pro-inflammatory/pro-repair model could not present the cell heterogeneity in vivo [26]. Therefore, we tried to explain the heterogeneity by functional enrichment of marker genes. Comparing the highly differentially expressed genes and function enrichments of each subtype (Fig. 2e and Additional file 7: Table S6), we found that scavenger receptors, such as MARCO and CD163 molecule (CD163), and complement receptors, such as complement C2 (C2), complement component 1, q subcomponent, alpha polypeptide (C1QA) and complement C1q B chain (C1QB) were highly expressed in cluster 0, indicating their phagocytic ability [27–31]. Versican (VCAN) was selectively expressed in cluster 1, which could promote the synthesis and secretion of inflammatory cytokines [32]. In the meanwhile, genes related to proinflammatory cytokines, including lysozyme (LYZ), S100A8 and S100 calcium binding protein A9 (S100A9) were also highly expressed in cluster 1[33, 34]. In contrast, genes related to adhesions and fibrosis such as secreted phosphoprotein 1 (SPP1) and CD9 molecule (CD9) were found at high levels in cluster 7 [35]. C–C motif chemokine ligand 2 (CCL2), C–C motif chemokine ligand 13 (CCL13), C–C motif chemokine ligand 18 (CCL18) and C-X-C motif chemokine ligand 12 (CXCL12) were highly expressed in cluster 3. Since CCL2 was the dominant chemokine gene for the migration of mononuclear phagocyte system and CCL13, CCL18 and CXCL12 were the critical chemokines, cluster 3 might play an important role in the chemotactic function [36]. Meanwhile, top differential genes also included apolipoprotein E (APOE), apolipoprotein C1 (APOC1) and legumain (LGMN) in this cluster, showing their ability of plasma lipoprotein regulation. Genes involved in class II antigen presentation were present at highest level in cluster 6, showing their functions in antigen processing and presentation. Importantly, we found two new subtypes of macrophages which were not reported previously in peritoneal fluid. One was cluster 11 which expressed high levels of TCRs (TRBC1 and TRBC2). The critical components of the TCR signal transduction machinery were also expressed in cluster 11, such as CD3D, CD3E, LCK proto-oncogene, Src family tyrosine kinase (LCK), zeta chain of T cell receptor associated protein kinase 70 (ZAP70), linker for activation of T cells (LAT) and FYN proto-oncogene, Src family tyrosine kinase (FYN), which were mostly matched with TCR^+^ macrophages [37, 38]. The other one was cluster 9 where genes associated with cell proliferation, including marker of proliferation Ki-67 (MKI67), cyclin dependent kinase 1 (CDK1), ubiquitin conjugating enzyme E2 C (UBE2C), baculoviral IAP repeat containing 5 (BIRC5) and KIAA0101, were highly and selectively expressed, indicating that the macrophages might be under the proliferating condition. Enriched GO analysis of each cluster supported these functions (Fig. 2e).

### Pseudo-time analysis exhibited differentiation trajectory of macrophages in peritoneal fluid

**Fig. 3:**
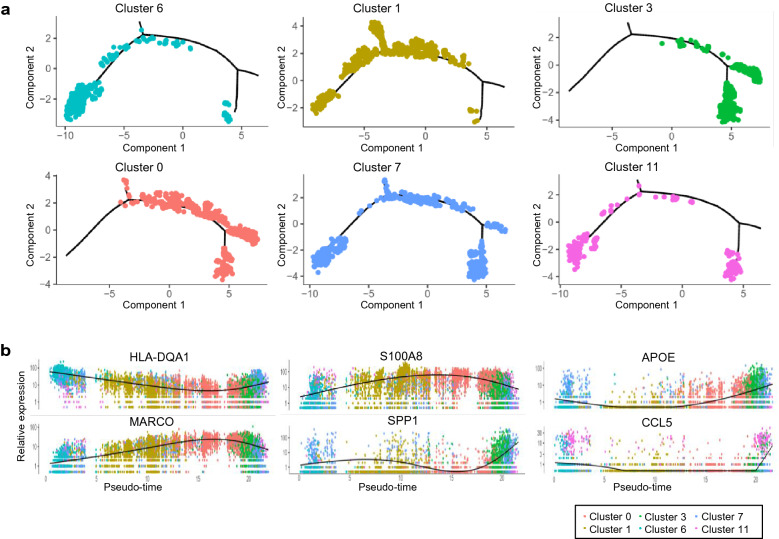
The differentiation trajectory of macrophages in peritoneal fluid by Pseudo-time analysis. **a** Pseudo-time of six subtypes of macrophages in peritoneal fluid inferred by Monocle package of R software. Each dot corresponds to one single cell. **b** Expressions of differential marker genes across six subtypes of macrophages, ordered by Monocle analysis in pseudo-time

To further investigate the differentiation trajectory of six clusters of macrophages (cluster 9 was excluded for the proliferating macrophages might not derived from monocytes), Monocle package of R software was applied for the analysis. The results showed that most cells from each cluster gathered based on the gene signatures and the six clusters formed into a relative process in pseudo-time. Specifically, it began with cluster 6 (antigen presentation) as they expressed the highest levels of CCR2 and CD33 which were the surface markers for monocytes (Additional file 1: Figure S2), followed by cluster 1 (pro-inflammatory), and ended with cluster 3 (chemotaxis) and cluster 0 (phagocytosis) (Fig. 3a, b). Furthermore, Cluster 7 (adhesion and fibrosis) were presented in the whole period of the pseudo-time but highly enriched at the late period, which indicated that macrophages also had an ability for tissue repair in the whole development process and this ability was enhanced at the final stage of differentiation. As the newly discovered subtype of macrophages, we discussed the functions of TCR^+^ macrophages below. Since retrograde menstruation was common in most women and macrophages were one of the main immune cells, the response of macrophages to the menstrual debris may follow the above differentiation trajectory after endometrial tissues invaded into the peritoneal cavity.

### Two newly discovered subtypes of macrophages in peritoneal fluid

**Fig. 4:**
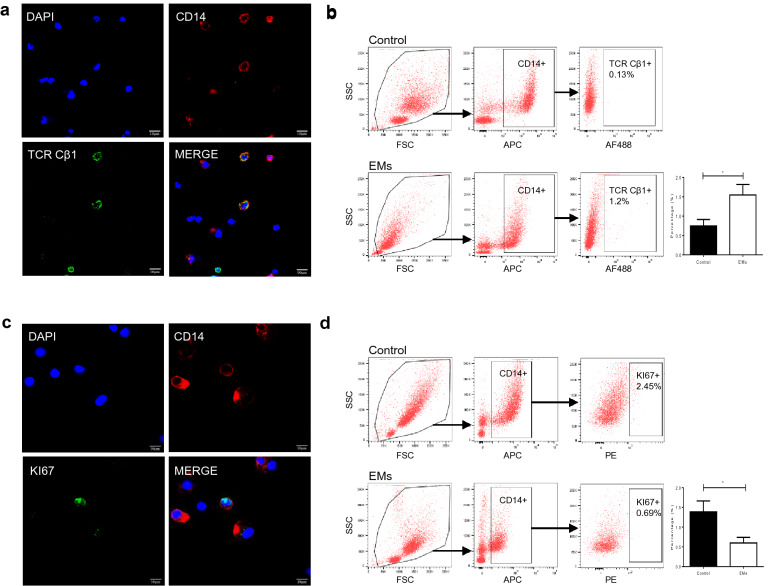
Two newly discovered subtypes of macrophages in peritoneal fluid. **a** Double immunofluorescence staining for CD14 (red) and TCR Cβ1 (green) antigens of ascites cell. Original magnification 1000×, scale bars = 10 µm. **b** The rate of TCR Cβ1 positive cells in isolated CD14 positive peritoneal macrophages tested by flow cytometric analysis. Bar plot shows the percentage of TCR Cβ1 positive macrophages in CD14 positive peritoneal macrophages isolated from the endometriosis (n = 13) and control samples (n = 9). **c** Double immunofluorescence staining for CD14 (red) and KI67 (green) antigens of ascites cell. Original magnification 1000×, scale bars = 10 µm. **d** The rate of KI67 positive cells in isolated CD14 positive peritoneal macrophages was tested by flow cytometric analysis. Bar plot shows the percentage of KI67 positive macrophages in CD14 positive peritoneal macrophages isolated from the endometriosis (n = 11) and control samples (n = 9). Data in **b** and **d** are presented as mean ± SEM. *EMs* endometriosis

As mentioned above, we discovered two new subtypes of macrophages in peritoneal fluid: TCR^+^ macrophages and proliferating macrophages. To confirm the existence of TCR^+^ macrophages in peritoneal fluid, double immunofluorescence staining of ascites cells was performed. We confirmed the marked existence of TCR Cβ1 (green) in CD14 (red) positive macrophages and the morphology of double positive cells looked as same as common macrophages and the size was bigger than that of T cells (Fig. 4a). Flow cytometry further confirmed that the percentage of TCR^+^ macrophages was elevated in endometriosis when compared to control (1.548 ± 0.271 vs. 0.747 ± 0.168, *P* = 0.0287) (Fig. 4b), which was consistent with the results tested by scRNA-seq. Granzyme (GZMK, GZMM, GZMH and GZMA) and immune mediators including C–C motif chemokine ligand 5 (CCL5) and C–C motif chemokine ligand 4 (CCL4) were highly expressed (Additional file 7: Table S6) in this cluster, indicating their cytotoxic and chemotactic effects. GO terms further revealed that they were enriched in granulocyte activation, cytokine production and response to bacterium (Fig. 2e).

The majority of macrophages in peritoneal fluid were derived from monocytes, which were terminally differentiated and did not have the ability of proliferation. However, proliferating macrophages (cluster 9) were discovered and they might be identified as tissue-resident macrophages [39]. We also verified the existence of this subtype of macrophages in peritoneal fluid using double immunofluorescence staining (Fig. 4c). Both scRNA-seq and flow cytometry revealed that the percentage of proliferating macrophages was decreased in endometriosis (0.5991 ± 0.142 vs. 1.388 ± 0.276, *P* = 0.0151) (Fig. 4d, Additional file 1: Figure S3).

### The dysfunction of macrophages in peritoneal fluid of endometriosis

**Fig. 5:**
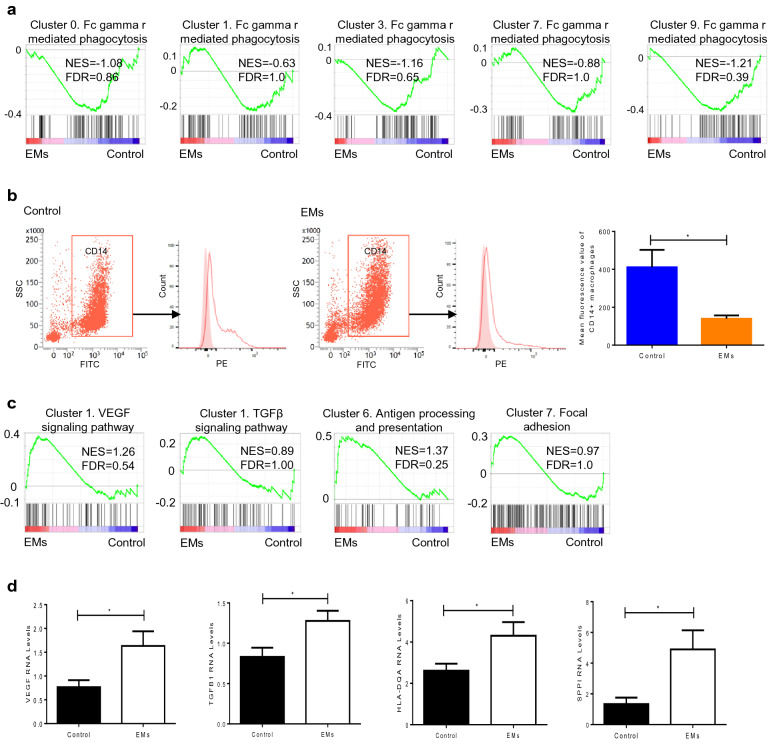
The dysfunction of macrophages in peritoneal fluid of endometriosis. **a** GSEA of Fc gamma r mediated phagocytosis pathway in different clusters of macrophages in endometriosis and control samples. **b** CD14+ macrophages phagocytosis was detected by flow cytometry in endometriosis and control groups. Light red and open red histograms correspond to background and fluorescence intensity of CD14+ macrophages with ingested bioparticles. Bar plot shows the mean fluorescence intensity of CD14+ macrophages in endometriosis group (n = 5) and control group (n = 5), *P* = 0.0200. Data are presented as mean ± SEM. **c** GSEA of the corresponding KEGG pathways in different clusters of macrophages in endometriosis and control groups. **d** The levels of mRNA expression for VEGF, TGFB1, HLA-DQA and SPPI of primary macrophages in peritoneal fluid from the endometriosis group (n = 10) and control group (n = 9). Data in **d** are presented as mean ± SEM. *EMs* endometriosis, *NES* normalized enrichment score, *FDR* false discovery rate, *GSEA* Gene Set Enrichment Analysis

In order to investigate if there was function deficiency of macrophages in peritoneal fluid of endometriosis, we applied GSEA to compare the differences between endometriosis and control samples. Cluster 0 which was the main subtype for phagocytosis had lower ability of phagocytosis in endometriosis. This dysfunction of phagocytosis could also be found in the other macrophage subtypes (Fig. 5a). Then we performed phagocytosis tests through flow cytometry to investigate the phagocytic ability of macrophages in peritoneal fluid. We found that the mean fluorescence intensity of CD14 + macrophages was significantly lower in endometriosis samples than that of control samples (139.6 ± 17.1 vs. 410.6 ± 92.0, *P* = 0.02) (Fig. 5b), indicating that the phagocytic ability of macrophages was decreased in peritoneal fluid of endometriosis. For the subtypes with pro-inflammation (cluster 1), antigen presentation (cluster 6) and adhesion and fibrosis (cluster 7), GSEA showed that the corresponding functions were elevated in endometriosis (Fig. 5c). Then, we sorted macrophages by flow cytometry and mRNA levels of functional genes were detected using Quantitative PCR analysis. We found the levels of corresponding functions genes were elevated in endometriosis groups compared to control groups (Fig. 5d), including VEGF (1.633 ± 0.308 vs. 0.765 ± 0.149, *P* = 0.0258), TGFB1 (1.277 ± 0.127 vs. 0.830 ± 0.115, *P* = 0.0192), HLA-DQA (4.304 ± 0.657 vs. 2.613 ± 0.339, *P* = 0.0410) and SPPI (4.896 ± 1.239 vs. 1.334 ± 0.422, *P* = 0.0187).These findings indicated that macrophages mainly had deficient ability for phagocytosis in endometriosis, which may be responsible for the incomplete clearance of refluxed menstrual debris.

### The cytotoxic activity of NK cells was decreased while chemotactic effect was elevated in peritoneal fluid of endometriosis

**Fig. 6:**
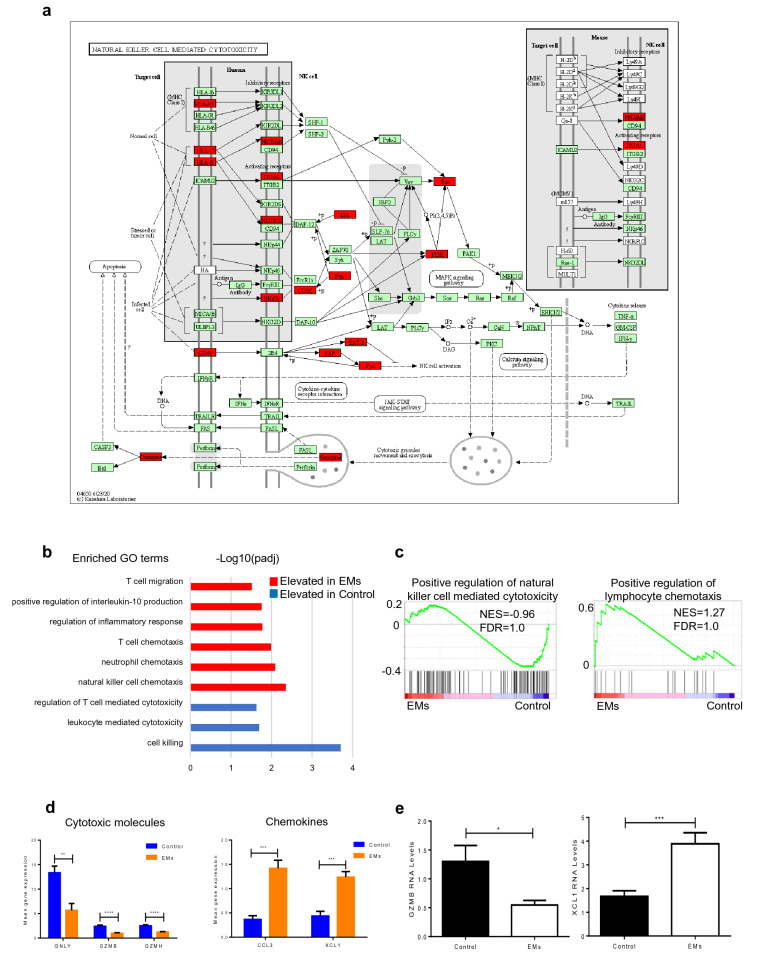
Dysfunction of NK cells in endometriosis. **a** Enriched KEGG pathway of natural killer cell mediated cytotoxicity of cluster 8. Red presents differentially expressed gene. **b** Enriched GO terms of natural killer cells. **c** Gene Set Enrichment Analysis of positive regulation of natural killer cell mediated cytotoxicity pathway and positive regulation of lymphocyte chemotaxis pathway of natural killer cells in endometriosis and control samples. **d** Mean gene expressions of GNLY, GZMB, GZMH, CCL3 and XCL1 of natural killer cells in endometriosis and control samples. **e** mRNA expressions of GZMB and XCL1 of primary NK cells in peritoneal fluid were detected by Quantitative PCR analysis for the endometriosis group (n = 9) and control group (n = 9). Data in Fig. 6d and Fig. 6e are presented as mean ± SEM. *EMs* endometriosis, *NES* normalized enrichment score, *FDR* false discovery rate

Of all the cell types detected, the number of NK cells was increased most significantly in endometriosis sample (5.26%) when compared to the control sample (1.99%) (Additional file 6: Table S5). Comparing the differential genes, we found that this cluster was enriched in the KEGG pathway of natural killer cell mediated cytotoxicity (Fig. 6a), which further confirmed the identification of cluster 8. Then, we focused on the function of NK cells in peritoneal fluid of endometriosis. Go enrichment analysis and GSEA revealed that the cytotoxic activity of NK cells was decreased in endometriosis while the pro-inflammatory and chemotactic effects were elevated (Fig. 6b, c). The highly differential genes between the two groups showed that C–C motif chemokine ligand 3 (CCL3) and X-C motif chemokine ligand 1 (XCL1) were significantly elevated in endometriosis while cytotoxic molecules including GNLY, GZMB and GZMH were significantly down-regulated (Fig. 6d). Then, we sorted NK cells by flow cytometry and mRNA levels of functional genes were measured by Quantitative PCR analysis. We found that the gene expression of GZMB was significantly down-regulated (0.546 ± 0.079 vs. 1.300 ± 0.278, *P* = 0.0149) while XCL1 was up-regulated (3.889 ± 0.466 vs. 1.672 ± 0.238, *P* = 0.0006) in endometriosis compared to that of control groups (Fig. 6e). These findings indicated that cytotoxic activity of NK cells was decreased while chemotactic effect was elevated in peritoneal fluid of endometriosis, which might play an important role in the pathology of endometriosis.

### Two subtypes of DCs in peritoneal fluid

**Fig. 7:**
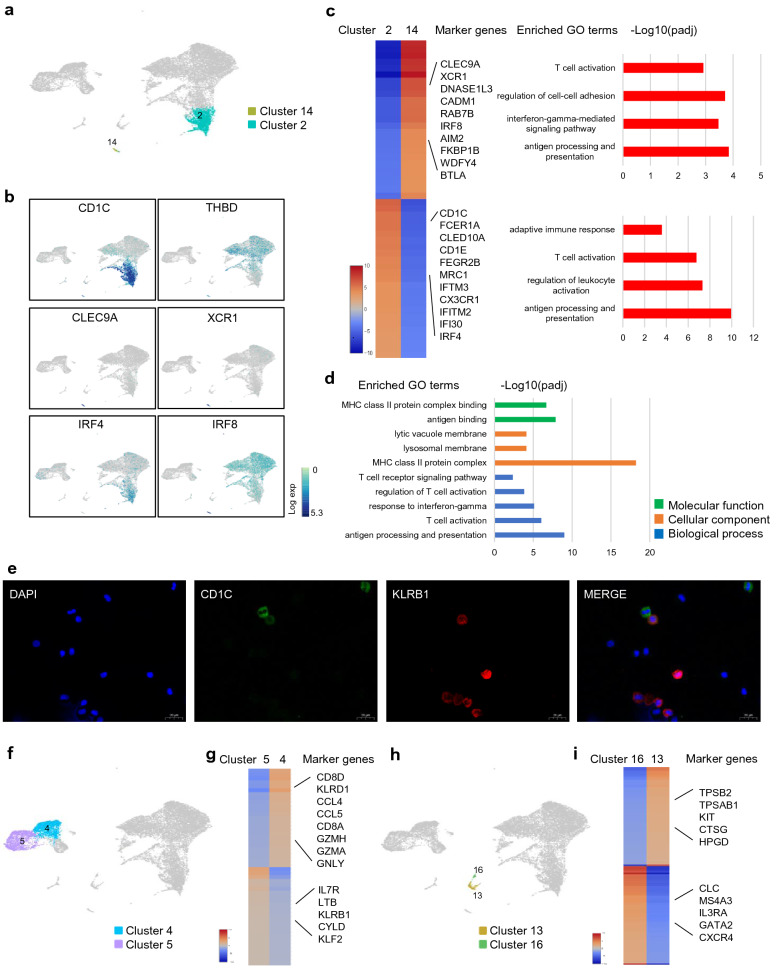
Dendritic cells, natural killer dendritic cells, T cells and mast cells in peritoneal fluid. **a**, **f**, **h** 2D visualization of clusters of dendritic cells (**a**), T cells (**f**) and mast cells (**h**) on the UMAP plots. Each dot corresponds to one single cell. **b** Distributions of CD1C, THBD, CLEC9A XCR1, IRF 4 and IRF 8 on the UMAP plots. **c** Heatmap, marker genes and enriched GO terms of dendritic cells. **d** Enriched GO terms of natural killer dendritic cells. **e** Double immunofluorescence staining for CD1C (green) and KLRB1 (red) antigens of ascites cells. Original magnification 400×, scale bars = 20 µm. **g, i** Heatmap and marker genes of T cells (**g**) and mast cells (**i**)

We identified two subtypes of DCs, cluster 2 and cluster 14 (Fig. 7a). Cluster 2 mapped closely to the well-established DC subtype of CD1C^+^ cDCs (Fig. 7b) [40]. Fc fragment of IgE receptor 1a (FCER1A), C-type lectin domain containing 10A (CLEC10A), mannose receptor C-type 1 (MRC1) and CD1E molecule (CD1E) were also highly expressed in this cluster (Fig. 7c and Additional file 8: Table S7). As for cluster 14, it mapped most closely to thrombomodulin^+^ (THBD^+^/CD141^+^) cDCs. But this commonly used marker (THBD) was a poor discriminator for this cluster, being also expressed by cells captured in macrophages (Fig. 7b). As C-type lectin domain containing 9A (CLEC9A) appeared to be a perfect discriminative marker gene for this cluster, we refer to this subtype as CLEC9A^+^ cDCs as previously reported [22]. In addition, X-C motif chemokine receptor 1 (XCR1) and deoxyribonuclease 1 like 3 (DNASE1L3) were also highly and selectively expressed in this cluster. We also detected transcription factors which played important roles in the development of DCs. We found that cluster 2 expressed high level of IRF 4, while cluster 14 expressed high levels of IRF 8, which was coincident with previous studies. Both of the DCs subtypes had the abilities of antigen uptake, presentation and leukocyte activation (Fig. 7c). CD1C^+^ cDCs were the main subtype of DCs in peritoneal fluid, which occupied 93.3% of the total DCs. On the other hand, CLEC9A^+^ cDCs showed the special capacity to induce CD8^+^ CTL responses with the high expression of CLEC9A and XCR1, the well-known receptors to cross-present antigens to CD8^+^ T cells [41, 42].

### NKDCs were firstly discovered in peritoneal fluid

As a rare cell type, NKDCs were not reported in peritoneal fluid previously, we investigated their differential marker genes and enriched functions. We found that NKDCs expressed high levels of DC markers (CD1C, ITGAX and CD1E) and MHC class II receptors (Additional file 5: Table S4), and also expressed NK cell markers (KLRB1, KLRD1 and NKG7) and T cell markers (CD3D, CD3E and CD3G) (Additional file 1: Figure S4). Enriched GO analysis revealed that NKDCs had the abilities of antigen processing and presentation, T cell activation and response to IFN-γ, indicating that NKDCs had abilities of both DCs and NK cells (Fig. 7d). These findings were consistent with previous reports [25]. Furthermore, we confirmed the existence of NKDCs in peritoneal fluid by double immunofluorescence staining (Fig. 7e).

### The dysfunction of T cells in peritoneal fluid of endometriosis

For T cells, we identified two subtypes (Fig. 7f). Cluster 4 mostly mapped the CD8^+^ T cells while cluster 5 mapped most closely to CD4^+^ T cells (Additional file 1: Figure S5a). KLRD1, CCL4, CCL5, GZMH, GZMA and granulysin (GNLY) were highly expressed in CD8^+^ T cells, indicating their cytotoxic and effector functions. The native markers, including interleukin 7 receptor (IL7R) and lymphotoxin beta (LTB), were expressed at high levels in CD4^+^ T cells (Fig. 7g and Additional file 9: Table S8). We further investigated the markers for regulatory T cells which was reported by previous literatures that played an important role in endometriosis [16, 43]. Unfortunately, these markers including forkhead box P3 (FOXP3), interleukin 2 receptor subunit alpha (IL2RA) and IKAROS family zinc finger 2 (IKZF2) expressed with low levels and did not form a cluster (Additional file 1: Figure S5b). We compared the functions of T cell between endometriosis and control sample as well. The number of T cells was elevated in endometriosis sample (Additional file 6: Table S5). However, the cytotoxic effect and chemotactic activity of T cells were dysfunctional in endometriosis by bioinformatic analysis (Additional file 1: Figure S5c).

### Mast cells and other cell types in peritoneal fluid

There were also two subtypes for mast cells (Fig. 7h). Cluster 13 seemed to be the activated mast cells as tryptase beta 2 (TPSB2), tryptase alpha/beta 1 (TPSAB1) which encoding tryptase were highly and selectively in this cluster. KIT proto-oncogene, receptor tyrosine kinase (KIT) was also highly expressed in cluster 13, which was another activating marker for mast cells (Fig. 7i and Additional file 10: Table S9) [44]. Cluster 16 might be a transition state from basophils as they expressed high levels of Charcot-Leyden crystal galectin (CLC), membrane spanning 4-domains A3 (MS4A3), interleukin 3 receptor subunit alpha (IL3RA) and GATA2 [45]. For plasma cells and other small number of cell types, we failed to conclude a significant functional difference during to the small number of cells.

## Discussion

Previous studies on the investigation of peritoneal fluid cell contents are mainly relied on flow cytometry or histological morphology [14, 46], while these techniques need prior knowledge and are limited to a small number of parameters. Here, we used scRNA-seq for the first time to investigate the cell contents and draw a comprehensive map of cell types in peritoneal fluid. We found that cells in peritoneal fluid were almost immune cells responsible for the clearance of refluxed menstrual debris and tissue defense. Macrophages are the largest immune population in peritoneal fluid, and followed by T cells and DCs, which is similar with previous studies [14, 46]. We also identified other groups of cells, including NK cells, mast cells, plasma cells and epithelial cells. Interestingly, we found an intermediate cell type which was named as NKDCs by Pillarisetty et al. [25]. We also found new cell subtypes of macrophages and revealed their functions by bioinformatic analysis as well. However, the complexity of the classification of cell groups was found in 10 × experiment, so the results should be treated with caution. Anyway, our study provides a fresh insight of peritoneal fluid cell contents and offers a useful resource for understanding menstruation and the pathology of endometriosis.

Our study consolidates and reinforces previous studies that immune dysfunction does existed in endometriosis [10]. As the first line of innate immunity, macrophages occupy the largest immune population in peritoneal fluid and have functional changes in endometriosis compared to control patients. The abilities of phagocytosis are defective in all seven subtypes which might lead to the incomplete clearance of refluxed menstrual debris and survival of endometrial cells. However, the pro-inflammatory, angiogenesis, adhesion and fibrosis effects are all elevated in endometriosis, which have been proved by previous studies [47]. The changed functions are also existed in other immune cells. NK cells are another main immune cell type to eliminate refluxed menstrual debris. Although the numbers of NK cells are elevated, the cytotoxic activity is found to be down-regulated in endometriosis. This decreased cytotoxic activity is also existed in T cells. These findings supported that immune dysfunction plays a central role in the development of endometriosis.

Therefore, we provide new insights into the peritoneal microenvironment in patients with advanced endometriosis and highlight several points of importance. Firstly, the peritoneal cavity is an immune microenvironment. The cells in peritoneal fluid are almost all immune cells responsible for the clearance of refluxed menstrual debris and infertility. Secondly, our results reveal that immune cells in peritoneal fluid, especially macrophages, are heterogeneous. Macrophages are the main immune cells in peritoneal fluid and might not comport with the pro-inflammatory/pro-repair polarization model. However, they might follow a certain differentiation trajectory after endometrial tissues invaded into the peritoneal cavity. Thirdly, the functions of immune cells in patients with endometriosis are defective. Generally speaking, the phagocytic and toxic effects of the immune cells are reduced while the pro-inflammatory and chemotactic effects are elevated. This immune dysfunction might play a central role in the pathology of endometriosis.

The present study has several limitations. Firstly, selection bias was inevitable because of small numbers of cases, although we selected the patient of advanced endometriosis with severe dysmenorrhea and the control patient without pelvic abnormalities verified by laparoscopic surgery. Secondly, our results might only reveal the peritoneal microenvironment of early proliferative phase as the cell contents and functions might change in different stage of menstrual cycle. Therefore, well-designed larger scale studies are required.

## Conclusion

Here, a comprehensive map of overall cells in peritoneal fluid was firstly exhibited by scRNA-seq. We provided a large-scale and high-dimensional characterization of peritoneal microenvironment and firstly reported several novel cell subtypes including TCR + macrophages, proliferating macrophages and natural killer dendritic cells in peritoneal fluid. The results also consolidate that immune dysfunction does existed in endometriosis and offer a useful resource for immunotherapy of endometriosis.

## Supplementary Information

**Additional file 1: Figure S1.** Diverse cell types in peritoneal fluid delineated by single cell transcriptomic analysis. **Figure S2.** Mean gene expressions of CCR2 and CD33 in the six macrophage subclusters. **Figure S3.** Gating strategy of flow cytometry for proliferating macrophages. **Figure S4.** Distributions of KLRB1, KLRD1, NKG7, CD3D, CD3E and CD3G on the UMAP plots. **Figure S5.** T cells in peritoneal fluid.

**Additional file 2: Table S1.** Clinical characteristics of endometriosis and control patients in this study.

**Additional file 3: Table S2.** Genes, UMIs counts, percentage of hemoglobin gene and percentage of mitochondrion for each individual cell.

**Additional file 4: Table S3.** The mean gene expression in each cluster of endometriosis (EMs) and control samples.

**Additional file 5: Table S4.** List of markers information for each cluster in ascites cells.

**Additional file 6: Table S5.** The numbers and percentages of each cell type in peritoneal fluid of endometriosis (EMs) and control samples.

**Additional file 7: Table S6.** List of differential genes in subtypes of macrophages.

**Additional file 8: Table S7.** List of differential genes in subtypes of dendritic cells.

**Additional file 9: Table S8.** List of differential genes in subtypes of T cells.

**Additional file 10: Table S9.** List of differential genes in subtypes of mast cells.

**Additional file 11: Table S10.** Details of antibodies used in this study.

**Additional file 12: Table S11.** Primer sequences for quantitative real-time PCR.

## Data Availability

All the data generated or analyzed during this study are included in this article and its supplementary files. The raw sequencing data have been deposited at NCBI’s Gene Expression Omnibus, with the Accession Number of PRJNA713993.

## Abbreviations

APOC1: Apolipoprotein C1
APOE: Apolipoprotein E
BIRC5: Baculoviral IAP repeat containing 5
C1QA: Complement component 1, q subcomponent, alpha polypeptide
C1QB: Complement C1q B chain
C2: Complement C2
CCL12: C–C motif chemokine ligand 12
CCL13: C–C motif chemokine ligand 13
CCL18: C–C motif chemokine ligand 18
CCL2: C–C motif chemokine ligand 2
CCL3: C–C motif chemokine ligand 3
CCL4: C–C motif chemokine ligand 4
CCL5: C–C motif chemokine ligand 5
CD14: CD14 molecule
CD163: CD163 molecule
CD1C: CD1C molecule
CD1E: CD1E molecule
CD3D: CD3D molecule
CD3E: CD3E molecule
CD3G: CD3G molecule
CD68: CD68 molecule
CD9: CD9 molecule
cDCs: Conventional dendritic cells
CDK1: Cyclin dependent kinase 1
CLC: Charcot–Leyden crystal galectin
CLEC9A: C-type lectin domain containing 9A
CLEC10A: C-type lectin domain containing 10A
CPA3: Carboxypeptidase A3
CXCL12: C-X-C motif chemokine ligand 12
DCs: Dendritic cells
DNASE1L3: Deoxyribonuclease 1 like 3
FCER1A: Fc fragment of IgE receptor 1a
FCGR3A/CD16: Fc fragment of IgG receptor IIIa
FOXP3: Forkhead box P3
FYN: FYN proto-oncogene, Src family tyrosine kinase
GATA2: GATA binding protein 2
GSEA: Gene Set Enrichment Analysis
GNLY: Granulysin
HLA-DRA: Histocompatibility complex, class II, DR alpha
IGHM: Immunoglobulin heavy constant mu
IKZF2: IKAROS family zinc finger 2
IL2RA: Interleukin 2 receptor subunit alpha
IL3RA: Interleukin 3 receptor subunit alpha
IL7R: Interleukin 7 receptor
ITGAX/CD11C: Integrin subunit alpha X
KIT: KIT proto-oncogene, receptor tyrosine kinase
KLRB1: Killer cell lectin like receptor B1
KLRD1: Killer cell lectin like receptor D1
KRT8: Keratin 8
LAT: Linker for activation of T cells
LCK: LCK proto-oncogene, Src family tyrosine kinase
LGMN: Legumain
LTB: Lymphotoxin beta
LYZ: Lysozyme
MARCO: Macrophage receptor with collagenous structure
MKI67: Marker of proliferation Ki-67
MRC1: Mannose receptor C-type 1
MS4A3: Membrane spanning 4-domains A3
MSigDB: Molecular Signatures Database
NCCRP1: NCCRP1, F-box associated domain containing
NK cells: Natural killer cells
NKDCs: Natural killer dendritic cells
NKG7: Natural killer cell granule protein 7
PCA: Principal component analysis
pDCs: Plasmacytoid dendritic cells
PTPRC/CD45: Protein tyrosine phosphatase receptor type C
S100A8: S100 calcium binding protein A8
S100A9: S100 calcium binding protein A9
scRNA-seq: Single-cell RNA-sequencing
SLCO5A1: Solute carrier organic anion transporter family member 5A1
SPP1: Secreted phosphoprotein 1
TCR: T cell receptor
THBD/CD141: Thrombomodulin
TPSAB1: Tryptase alpha/beta 1
TPSB2: Tryptase beta 2
Treg: Regulatory T cells
UMAP: Uniform manifold approximation and projection
UMIs: Unique molecule identifiers
UBE2C: Ubiquitin conjugating enzyme E2 C
VCAN: Versican
XCL1: X-C motif chemokine ligand 1
XCR1: X-C motif chemokine receptor 1
ZAP70: Zeta chain of T cell receptor associated protein kinase 70
