# Supplementary material for: Cell subtypes and immune dysfunction in peritoneal fluid of endometriosis revealed by single-cell RNA-sequencing
Source: Cell Biosci. 2021 May 26;11:98. doi: 10.1186/s13578-021-00613-5 (PMC8157653; doi:10.1186/s13578-021-00613-5)
Supplement: Supplementary file 1 — Additional file 1: Figure S1. Diverse cell types in peritoneal fluid delineated by single cell transcriptomic analysis. Figure S2. Mean gene expressions of CCR2 and CD33 in the six macrophage subclusters. Figure S3. Gating strategy of flow cytometry for proliferating macrophages. Figure S4. Distributions of KLRB1, KLRD1, NKG7, CD3D, CD3E and CD3G on the UMAP plots. Figure S5. T cells in peritoneal fluid. [file 13578_2021_613_MOESM1_ESM.docx]

**Additional file 1：**


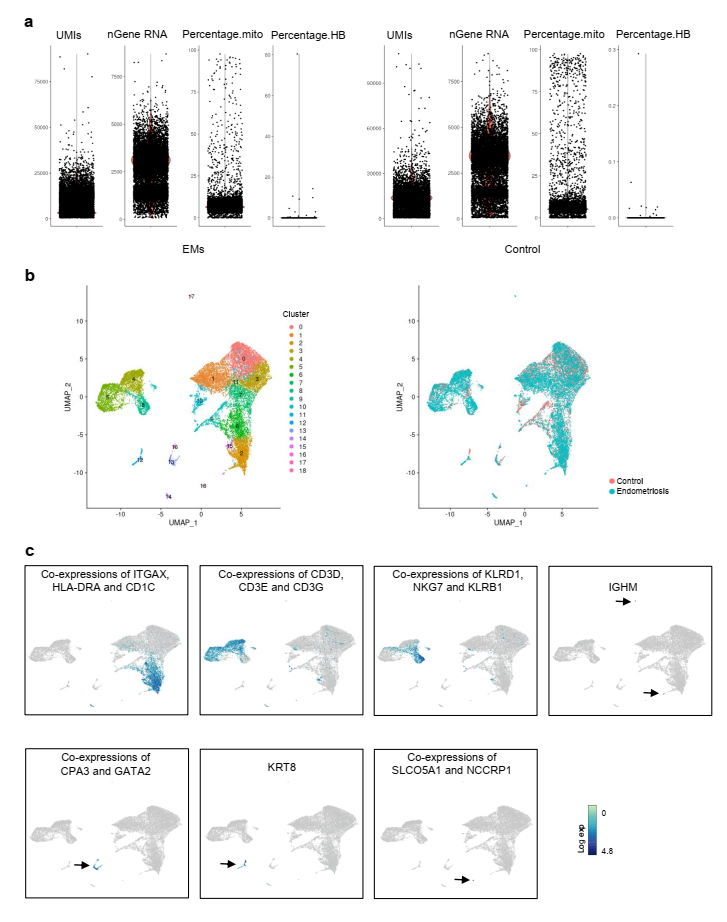


**Figure S1** Diverse cell types in peritoneal fluid delineated by single cell transcriptomic analysis. **a** Genes, UMIs, percentage of mitochondrion and percentage of hemoglobin gene for each individual cell of endometriosis and control sample. **b** 2D visualization of 19 clusters and two groups on the UMAP plots. Each dot corresponds to one single cell, colored according to cluster and location. **c** Co-expressions of certain marker genes across 17,530 single cells illustrated on the UMAP plots. The black arrow figures out the positive cells. EMs, endometriosis; UMIs, unique molecular identifiers

**Figure S2** Mean gene expressions of CCR2 and CD33 in the six macrophage subclusters.


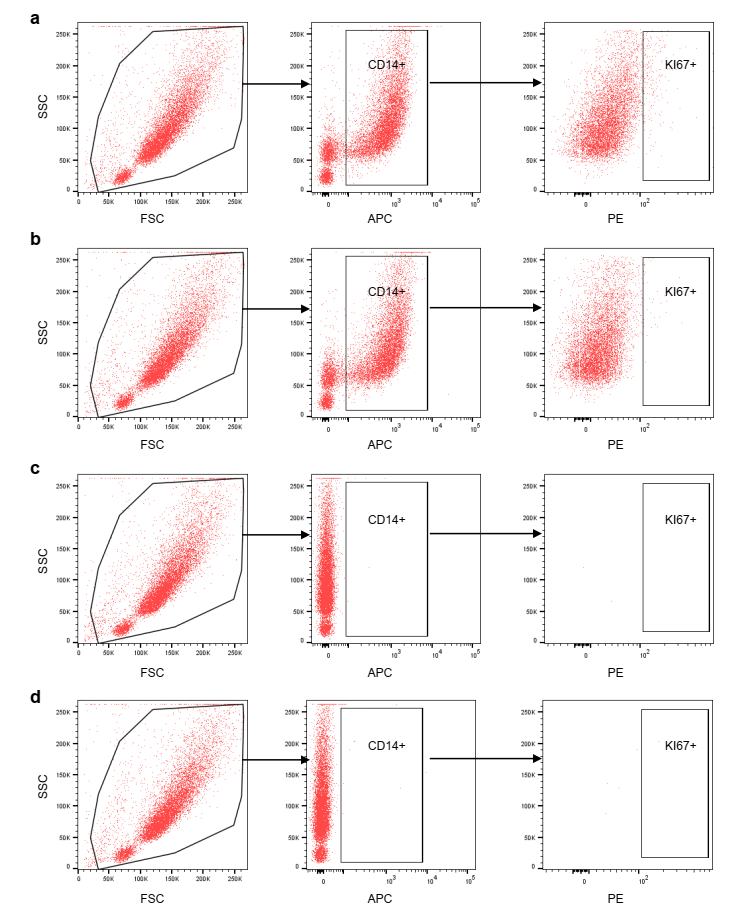


**Figure S3** Gating strategy of flow cytometry for proliferating macrophages. **a** Double staining for CD14 and KI67. **b** Staining for CD14 and KI67 isotype control. **c** Staining for CD14 isotype control and KI67. **d** Staining for CD14 isotype control and KI67 isotype control.


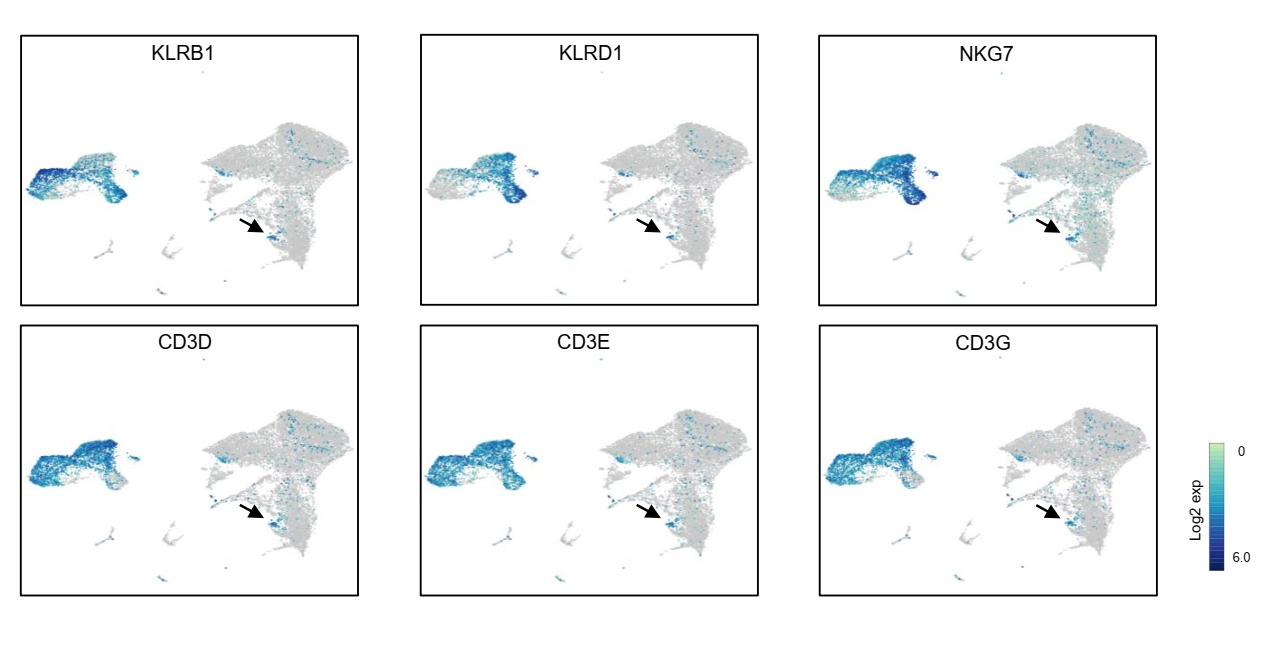


**Figure S4** Distributions of KLRB1, KLRD1, NKG7, CD3D, CD3E and CD3G on the UMAP plots. Black arrow figures out the positive cells in natural killer dendritic cells


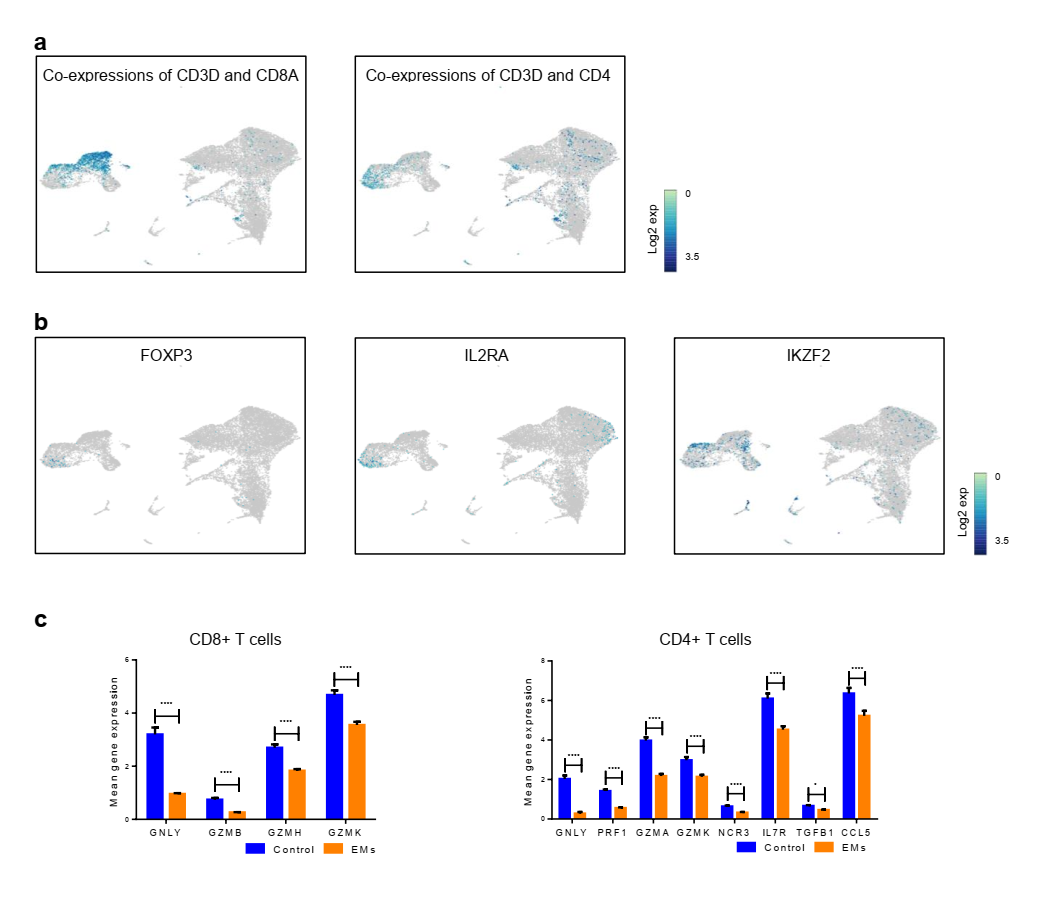


**Figure S5** T cells in peritoneal fluid. **a** Co-expressions of CD3D and CD8A on the UMAP plot. Co-expressions of CD3D and CD4 on the UMAP plot. **b** Distributions of FOXP3, IL2RA and IKZF2 on the UMAP plots. **c** Mean gene expressions of GNLY, GZMB, GZMH and GZMK of CD8^+^ T cells in endometriosis and control samples. Mean gene expressions of GNLY, PRF1, GZMA, GZMK, NCR3, IL7R, TGFB1, CCL4 and CCL5 of CD4^+^ T cells in endometriosis and control samples. EMs, endometriosis
