# Supplementary material for: Cell subtypes and immune dysfunction in peritoneal fluid of endometriosis revealed by single-cell RNA-sequencing
Source: Cell Biosci. 2021 May 26;11:98. doi: 10.1186/s13578-021-00613-5 (PMC8157653; doi:10.1186/s13578-021-00613-5)
Supplement: Supplementary file 11 — Additional file 11: Table S10. Details of antibodies used in this study. [file 13578_2021_613_MOESM11_ESM.docx]

**Table S10. Details of antibodies used in this study. Related to experimental procedures.** Working volume represent the volume of the antibody added per 100 µl of staining buffer.

| Marker | Working volume | Clone | Conjugate | Company | Cattalog# | Location |
| --- | --- | --- | --- | --- | --- | --- |
| CD14 | 5 µL | 61D3 | APC | eBioscience | 17-0149-42 | Cell surface |
| CD14 | 20 µL | MEM-15 | FITC | Abcam | Ab28061 | Cell surface |
| KLRB1 | 5 µL | HP-3G10 | APC | eBioscience | 17-1619-42 | Cell surface |
| KLRD1 | 5 µL | DX22 | PE | eBioscience | 12-0949-42 | Cell surface |
| CD1C | 5 µL | L161 | PE | eBioscience | 12-0015-42 | Cell surface |
| TCR Cβ1 | 5 µL | JOVI.1 | AF 488 | Santa | sc-53196 | Cell surface |
| KI67 | 20 µL | B56 | PE | BD | 556027 | Intracellular |
