# Supplementary material for: Cell subtypes and immune dysfunction in peritoneal fluid of endometriosis revealed by single-cell RNA-sequencing
Source: Cell Biosci. 2021 May 26;11:98. doi: 10.1186/s13578-021-00613-5 (PMC8157653; doi:10.1186/s13578-021-00613-5)
Supplement: Supplementary file 12 — Additional file 12: Table S11. Primer sequences for quantitative real-time PCR. [file 13578_2021_613_MOESM12_ESM.docx]

**Table S11. Primer sequences for quantitative real-time PCR.**

| Primer | Forward (5’-3’) | Reverse (3’-5’) |
| --- | --- | --- |
| SPP1 | TGAAACGAGTCAGCTGGATG | TGAAATTCATGGCTGTGGAA |
| HLA.DQA1 | TGGGCAGTCAGTCACAGAAG | GGCTCCCAGTGTTTCAGAAG |
| VEGF | CCCTTGACTGTGGAGCTCAT | GGCTTCACAGCACTGTCCTT |
| TGFB1 | CTTTCCTGCTTCTCATGGCC | TCCAGGCTCCAAATGTAGGG |
| GZMB | TGACAGTGCAGGAAGATCGA | ATAGGAGACAATGCCCTGGG |
| XCL1 | GACTGCCGGTTAGCAGAATC | GTTGGCTTGGTCTGGATCAT |
